# Nutritional knowledge, attitude and practice toward micronutrients among Iranian households: the NUTRI-KAP survey

**DOI:** 10.1186/s40200-016-0260-8

**Published:** 2016-10-04

**Authors:** Ramin Heshmat, Zahra Abdollahi, Farzaneh Sadeghi Ghotbabadi, Mahsa Rostami, Gita shafiee, Mostafa Qorbani, Mohsen Rezaei Homami, Bagher Larijani, Forouzan Salehi

**Affiliations:** 1 Chronic Diseases Research Center, Endocrinology and Metabolism Population Sciences Institute, Tehran University of Medical Sciences, Tehran, Iran; 2 Community Nutrition Department, Ministry of Health and Medical Education, Tehran, Iran; 3 Endocrinology & Metabolism Research Center, Endocrinology and Metabolism Clinical Sciences Institute, Tehran University of Medical Sciences, Tehran, Iran; 4 Department of Community Medicine, Alborz University of Medical Sciences, Karaj, Iran; 5 Rasoul-e-Akram Hospital Complex, Iran University of Medical Sciences, Tehran, Iran

**Keywords:** Nutrition, Knowledge, Attitude, Practice

## Abstract

**Background:**

Healthy diet for maintaining a healthy weight and prevention of chronic disease is vital at all stages of life. The purpose of this study was to determine the nutritional knowledge, attitude, and practice among urban and rural households in Iran.

**Methods:**

In this nation-wide study, 14,136 subjects were selected using a multistage cluster sampling method from 31 provinces of Iran. This study was on the households in rural and urban areas in Iran. Participants of this study were mothers or other members who were responsible for preparing meals for the whole family. Data were gathered by the questionnaire and the interview with the qualified person in each family.

**Results:**

Frequency of knowledge about food source of calcium was between 11.6 and 64.7 %. Knowing of food source of zinc was about 12.8–16.7 %. Knowledge about of meat as source of iron was 50.9 and 46.5 % in regions of urban and rural, respectively. Positive attitude about preferring to use the full-fat dairy was about 25.1 % and negative attitude about this issue was 71.4 %; the positive attitude was slightly lower in rural areas than in urban areas. Respectively, frequency of using red meat in urban households was 10.8, 73.7, and 15.5 % as daily, weekly and rarely. Frequency of using daily of milk, yogurt and cheese was significantly lower in rural than in urban household.

**Conclusion:**

This national study indicates that in some cases there is a gap between knowledge and behavior among household of Iranian population.

## Background

Recently, changes in lifestyle have led to an increase in the rate of chronic diseases such as cardiovascular diseases, stroke, osteoporosis, cancer, hypertension, and diabetes [1]. Studies have shown inappropriate feeding practices can be a cause of increasing prevalence of disease and other problems [2–4]. Micronutrients plays a main role in metabolism of cells and health [5]. Micronutrients are vital nutrients and these are found in vitamins, minerals and trace elements [6].

It seems appropriate nutrition intervention can be important in the choice of food. The factors related with food choices among individuals are nutrition knowledge, attitude and practice [7, 8]. Studies in Iran showed 75.5 % of households in intake of calcium, 43.9 % of them in intake of iron, and 41.7 % in intake of vitamin A, 39.1 % in intake of riboflavin and 30.9 % in intake of vitamin C were deficient. A study in Iran have shown that daily nutritional in university students have some deficiency in calcium, vitamins A, C and Iron for female [9]. Knowledge of nutrition is introduced to decrease of some diseases [10, 11]. It seems malnutrition is generally due to lack of nutritional information rather food deficiency. Therefore Education can improve nutritional knowledge. On the other hand, the majority of nutritional practices do not accord with nutritional knowledge. Studies have shown there is a gap between knowledge and Performance [12]. Since studies regarding of micronutrients among Iranian households has not been done, so study in this field is essential. The purpose of this research was to determine the knowledge, attitude and practice (KAP) of households about micronutrients in urban and rural regions of Iran.

## Methods

### Subjects and sampling

The participants of present study were 14,136 people who were responsible for preparing meals for the whole family and were older than 15 years old. Participants were selected from households from rural and urban areas in 31 provinces of Iran through multi-single cluster sampling technique. Only Iranian households were included in this study and foreign families were excluded, also if a family was absent at the time of interview for three times was excluded from the study. The protocol of the study was published previously [13, 14].

### Data collection

Data were gathered by a structured questionnaire and interview with qualified person in each family. The validity and reliability of the questionnaire was assessed in the pilot study and Cronbachs alpha was reported 0.79 [13]. KAP of Iranian households were assessed about micronutrients including calcium, zinc, Iron, and vitamin D. The knowledge grade was measured by 5 items, and categories of response were she/he knows or she/he does not know. Status of attitude was assessed by 5 items and categories of response were from 1 = I completely agree to 5 = I completely disagree. By 11 items, practice status was determined and answers ranged from daily to never.

After completion of the sampling questionnaires were controlled by officers in each province. If there were imperfectly or incompletely questionnaires, they were returned to the interviewees for further clarify and correction of any missing or inconsistent data. Also, randomly 10 % of the questionnaires of each province for identify any mistake were rechecked.

### Statistical analysis

Data were analyzed by the Statistical Package for the Social Sciences software version 16 (SPSS Inc., Chicago, IL, USA). Descriptive variables were reported as frequency (%95 Confidence Interval). Method of sampling (cluster sampling) was considered in all statistical analysis. The significant level was *P*-value < 0.05.

## Result

Table 1 shows the knowledge of Iranian households about micronutrient based on regions. Totally, frequency of knowledge about food source of calcium was between 11.6 % (CI 95 %: 10.7–12.5) and 64.7 % (CI 95 %: 63.3–66.1) among Iranian households. Significantly, urban families had more information about food source of calcium compared to rural households (*P*-value < 0.001). Knowledge about of food source of zinc was about 12.8–16.7 % in Iranian population, and there was significant different about knowledge of this issue between urban and rural areas. Respectively, knowledge about of zinc as nutrient was 34.7 % (CI 95 %: 33.2–36.2) and 18.6 % (CI 95 %: 16.9–20.5) in urban and rural regions. Knowledge about of meat as source of iron was 50.9 % (CI 95 %: 49.5–52.4) and 46.5 % (CI 95 %: 44.3–48.8) in urban and rural areas, respectively.

**Table 1:** Correct knowledge of Iranian households about micronutrient based on Region: the NUTRI-KAP survey

		Urban	Rural	Total	*P*-value
Knowledge about of food source of calcium	Milk	67.9* (66.3–69.6)	59.0 (56.4–61.4)	64.7 (63.3–66.1)	<0.001
	Yogurt	59.7 (58.1–61.4)	50.6 (48.1–53.1)	56.5 (55.1–57.9)	<0.001
	Cheese	48.3 (46.6–50.1)	39.8 (37.4,42.2)	45.3 (43.9–46.7)	<0.001
	Yogurt	36.9 (35.1–38.7)	31.4 (29.1–33.9)	34.9 (33.5–36.4)	<0.001
	Curd	29.1 (27.4,30.8)	22.8 (20.6–25.1)	26.8 (25.5–28.2)	<0.001
	Types of Cabbage	13.6 (12.5–14.8)	7.9 (6.7–9.2)	11.6 (10.7–12.5)	<0.001
Knowledge about of the best source of vitamin D (Direct sunlight)		33.1 (31.7–34.5)	17.7 (16.1–19.4)	27.7 (26.6–28.8)	<0.001
Knowledge about of zinc as a nutrient		34.7 (33.2–36.2)	18.6 (16.9–20.5)	29.0 (27.9–30.2)	<0.001
Knowledge about of food source of zinc	Meat	16.6 (15.1–18.3)	16.9 (13.9–20.3)	16.7 (15.2–18.2)	0.9
	liver	8.4 (7.1–9.8)	13.3 (10.8–16.4)	9.6 (8.4–10.9)	<0.001
	Cheese	3.4 (2.8–4.1)	8.7 (6.2–11.9)	4.7 (3.9–5.7)	<0.001
	Nuts	13.3 (11.8–14.9)	11.3 (9.0–13.9)	12.8 (11.5–14.1)	0.2
Knowledge about of food source of iron	Meat	50.9 (49.5–52.4)	46.5 (44.3–48.8)	49.4 (48.2–50.1)	<0.001
	liver	39.8 (38.1–41.4)	32.2 (29.8–34.7)	37.1 (35.7–38.5)	<0.001
	Eggs	17.8 (16.6–19.0)	18.6 (16.9–20.4)	18.1 (17.1–19.1)	0.4
	bean	47.4 (45.8–48.9)	45.7 (43.4–48.0)	46.8 (45.5–48.1)	0.2
	Vegetables	56.3 (54.7–57.8)	45.5 (43.2–47.8)	52.4 (51.1–53.7)	<0.001
	Dried fruit and nuts	18.6 (17.4–20.0)	15.5 (13.8–17.3)	17.5 (16.5–18.6)	0.006

*% (95 % CI), *P*-value <0.05

Table 2 shows the attitude of Iranian households about micronutrient based on regions. Positive attitude about preferring to use the full-fat dairy was about 25.1 % (CI 95 %: 24.0–26.3) and negative attitude about this issue was 71.4 % (CI 95 %: 70.2–72.6), which positive attitude was slightly lower in rural than urban areas (*P*-value <0.001). Significantly, positive attitude about keeping children out of direct sunlight in rural regions was slightly higher than urban regions (27.5 % vs. 32.8 %, *P*-value < 0.001). Respectively, favorable attitude about the same nutritional value of mushrooms as meat in urban and rural families were 9.6 % (CI 95 %: 8.8–10.4), and 13.4 % (CI 95 %: 11.9–14.9).

**Table 2:** Attitude of Iranian households about micronutrient based on Region: the NUTRI-KAP survey

		Urban	Rural	Total	*P*-value
Preferring to use the full-fat dairy	I agree	22.83* (21.5–24.1)	29.3 (27.4–31.3)	25.1 (24.0–26.3)	<0.001
	I have no idea	3.5 (2.9–4.3)	3.4 (2.6–4.4)	3.5 (2.9–4.1)	<0.001
	I disagree	73.6 (72.2–75.1)	67.3 (65.1–69.4)	71.4 (70.2–72.6)	<0.001
Keeping children out of direct sunlight	I agree	27.5 (26.1–28.9)	32.8 (30.9–34.8)	29.4 (28.2–30.6)	<0.001
	I have no idea	2.6 (2.2–3.0)	3.4 (2.8–4.1)	2.9 (2.5–3.2)	<0.001
	I disagree	69.9 (68.4–71.4)	63.8 (61.7–65.8)	67.7 (66.5–68.9)	<0.001
Daily milk intake only for children is required.	I agree	10.4 (9.5–11.5)	12.4 (10.9–13.9)	11.1 (10.3–12.0)	0.02
	I have no idea	0.7 (0.5–0.9)	0.95 (0.68–1.31)	0.77 0.62–0.95	0.02
	I disagree	88.9 (87.8–89.9)	86.7 (84.9–88.2)	88.1 (87.2–88.9)	0.02
Benefits of chocolate milk consumption in children who do not drink milk.	I agree	42.1 (40.5–43.6)	40.2 (38.2–42.3)	41.4 (40.2–42.7)	0.3
	I have no idea	13.8 (12.7–15.0)	16.3 (14.7–18.1)	14.7 (13.7–15.7)	0.3
	I disagree	44.2 (42.6–45.7)	43.5 (41.5–45.5)	43.9 (42.7–45.1)	0.3
The same nutritional value of mushrooms as meat	I agree	65.1 (63.6–66.6)	60.9 (58.7–63.1)	63.6 (62.4–64.85	<0.001
	I have no idea	9.6 (8.8–10.4)	13.4 (11.9–14.9)	10.9 (10.2–11.7)	<0.001
	I disagree	25.3 (23.9–26.7)	25.8 (23.9–27.6)	25.5 (24.4–26.9)	<0.001

*%(95 % CI), *P*-value <0.05

Table 3 shows practice of Iranian households about consumption of food source of micronutrient. Totally, frequency of red meat consumption among Iranian population was 10.1 % (CI 95 %: 9.3–10.9), 71.1 % (CI 95 %:69.9–72.2), and 18.9 % (CI 95 %: 17.8–19.9) as daily, weekly and rarely, respectively. Significantly, red meat consumption was higher in urban households than rural households (*P*-value <0.001). The rate of fish intake among Iranian population was 1.9 % (CI 95 %: 1.5–2.2) and 40.3 % (CI 95 %: 38.9–41.6), and 57.9 % (CI 95 %: 56.5–59.3) as daily, weekly and rarely, respectively. Frequency of weekly consumption of fish in rural households (2.5 %, CI 95 %: 1.8–3.4) was significantly higher than urban households (1.5 %, CI 95 %: 1.5–2.3). Frequency of daily consumption of fresh vegetables in urban households (59.3 %, CI 95 %: 57.6–61.0) was significantly greater than rural households (43.7 %, CI 95 %: 41.1–46.3). Frequency of daily intake of milk, yogurt and cheese in rural households was significantly lower than urban household (78.6 % vs. 84.8, *P*-value < 0.001).

**Table 3:** Practice of Iranian households about micronutrient based on Region: the NUTRI-KAP survey

		Urban	Rural	Total	*P*-value
Frequency of red meat consumption in your households	Daily	10.8* (9.8–11.8)	8.8 (7.6–10.2)	10.1 (9.3–10.9)	<0.001
	Every Week	73.7 (72.4–75.1)	66.2 (64.0–68.2)	71.1 (69.9–72.2)	<0.001
	Rarely	15.5 (14.4–16.7)	25.0 (23.1–27.1)	18.9 (17.8–19.9)	<0.001
Frequency of Liver, heart and kidneys consumption in your households	Daily	0.98 (0.72–0.37)	0.40 (0.26–0.64)	0.78 (0.59–1.03)	0.06
	Every Week	17.8 (16.6–19.1)	18.8 (17.2–20.4)	18.1 (17.2–19.2)	0.06
	Rarely	81.2 (79.9–82.5)	80.83 (79.2–82.4)	81.1 (80.0–82.1)	0.06
Frequency of chicken and cygnus consumption in your households	Daily	12.4 (11.3–13.6)	11.6 (10.1–13.3)	12.13 (11.23–13.1)	0.06
	Every Week	78.8 (77.4–80.1)	77.7 (75.8–79.6)	78.4 (77.3–79.5)	0.06
	Rarely	8.8 (8.04–9.59)	10.7 (9.5–12.0)	9.5 (8.8–10.1)	0.06
Frequency of fish consumption in your households	Daily	1.5 (1.5–2.3)	2.5 (1.8–3.4)	1.9 1.518,2.27	<0.001
	Every Week	43.6 (42.0–45.2)	34.3 (32.0–36.6)	40.3 (38.9–41.6)	<0.001
	Rarely	54.9 (53.3–56.6)	63.2 (60.7–65.6)	57.9 (56.5–59.3)	<0.001
Frequency of eggs consumption in your households	Daily	23.5 (22.2–24.9)	26.8 (24.8–29.0)	24.7 (23.6–25.8)	0.004
	Every Week	67.0 (65.5–68.5)	62.8 (60.6–64.9)	65.51 (64.3–66.7)	0.004
	Rarely	9.5 (8.7–10.4)	10.4 (9.2–11.7)	9.8 (9.1–10.5)	0.004
Frequency of bean consumption your households	Daily	19.9 (18.6–21.3)	19.4 (17.6–21.4)	19.8 (18.7–20.9)	0.03
	Every Week	71.5 (70.0–73.0)	73.9 (71.8–75.8)	72.4 (71.1–73.5)	0.03
	Rarely	8.5 (7.7–9.4)	6.7 (5.8–7.71)	7.9 (7.3–8.5)	0.03
Frequency of fresh vegetables consumption (salad and fresh herbs) your households	Daily	59.3 (57.6–61.0)	43.7 (41.1,46.3)	53.8 (52.3–55.2)	<0.001
	Every Week	34.7 (33.2–36.3)	45.1 (42.7–47.4)	38.4 (37.1–39.7)	<0.001
	Rarely	6.0 (5.3–6.7)	11.3 (9.96–12.7)	7.9 (7.2–8.6)	<0.001
Frequency of milk, yogurt and cheese consumption your households	Daily	84.8 (83.6–86.0)	78.6 (76.3–80.7)	82.6 (81.5–83.7)	<0.001
	Every Week	12.5 (11.5–13.6)	18.3 (16.4–20.4)	14.6 (13.6–15.6)	<0.001
	Rarely	2.7 (2.2–3.2)	3.2 (2.6–3.9)	2.8 (2.5–3.3)	<0.001
Frequency of butter, cream or cream consumption in your households	Daily	28.3 (27.0–29.7)	30.3 (28.3–32.3)	29.0 (27.9–30.1)	0.3
	Every Week	38.4 (36.9–39.8)	37.2 (35.2–39.2)	38.0 (36.8–39.1)	0.3
	Rarely	33.3 (31.9–34.8)	32.6 (30.5–34.6)	33.1 (31.9–34.3)	0.3
Frequency of nuts and dried fruits consumption in your households	Daily	16.2 (15.1–17.43)	16.9 (15.4–18.6)	16.5 (15.6–17.4)	0.5
	Every Week	33.4 (32.0–34.9)	31.9 (30.0–33.9)	32.9 (31.8–34.1)	0.5
	Rarely	50.3 (48.7–52.0)	51.2 (49.0–53.4)	50.6 (49.3–52.0)	0.5
Frequency of doogh consumption in your households	Daily	39.9 (38.3–41.6)	44.6 (42.2–47.1)	41.6 (40.2–43.0)	0.001
	Every Week	47.8 (46.2–49.4)	43.0 (40.8–45.2)	46.1 (44.8–47.4)	0.001
	Rarely	12.3 (11.3–13.2)	12.4 (11.1–13.8)	12.3 (11.5–13.1)	0.001

*%(95 % CI), *P*-value <0.05

## Discussion

Healthy diet plays a vital role in the country’s human resource development. Past studies have shown which determination of knowledge, attitude and practice of the population for improvement of health program is essential [15]. In our study, the KAP of Iranian households toward micronutrients including zinc, iron, calcium, and vitamin D were assessed in urban and rural regions.

Our data showed knowledge about food sources of micronutrients among urban households were significantly higher than rural households. Likely, this difference was caused by information poverty among villagers. So we should pay attention to this issue which peoples in rural area do not enough information about micronutrients.

Past study indicated there is an association between nutrition knowledge and healthy diet. In addition, nutritional knowledge has the potential to contribute to improving dietary quality [7]. The findings of our study showed that knowledge level of urban and rural families about food source of calcium, zinc, and direct sunlight as best source of vitamin D were relatively low. So, enhancing of the training program is offered in both areas.

In this study positive attitude about the same nutritional value of mushrooms as meat was 63.6 % and negative attitude was about 25.5 %. Favorable attitude about preferring to use the full-fat dairy was 22.8 % and 29.3 % among urban and rural households, respectively. Past survey of attitude and beliefs about diet and nutrition in some countries of the European union found which there was difference eating patterns among various cultural [16]. In addition, another study in china has shown although majority of the residents understand about unhealthy foods but only a few of them take action to healthy foods intake [17]. Behavior of an individual is determined by person attitude and community pressure.

Our data demonstrated that performance of nutrition was different in urban and rural areas in Iran. Our study showed that frequency of daily consumption of red meat in Iranian households was 10.8 %, and 8.8 % and frequency of weekly intake was 73.7 % and 66.2 % in urban and rural regions, respectively. So, a significant difference was observed between these regions (<0.001). Also frequency of daily intake of fish was about 12.4 % in urban and 11.6 % in rural areas. And frequency of weekly using was 78.8 % and 77.7 % in urban and rural regions, respectively. Frequency of use of milk, yogurt and cheese was 84.8 and 78.6 % among urban and rural households, respectively. The study among women in Tabriz showed despite high levels of awareness but performance was poor [18]. Influential factors on practice can be beliefs, availability of food and economics issues. Changing households’ attitudes, knowledge and awareness about healthy diet leads them to make better food choices. Past studies have shown that poor nutritional knowledge may lead to inappropriate nutritional practice [19]. Nevertheless, there are different issues which lead to the distance between knowledge and practice. So it is essential that these factors should be identified and resolved at the community.

## Conclusion

This national study showed that KAP of nutrition was different in urban and rural areas. And also generally in some cases there are distance between knowledge and behavior. It seems we need to pay attention to this regard more in order to increase KAP of community and increase of healthy diet among urban and rural community.

## Abbreviation

KAP: Knowledge, attitude and practice

## References

[1] Shimizu T (2003). Health Claims and Scientific Substantiation of Functional Foods-Japanese System Aiming the Global Standard. Curr Top Nutraceutical Res..

[2] Kant AK (2000). Consumption of energy-dense, nutrient-poor foods by adult Americans: nutritional and health implications. The third National Health and Nutrition Examination Survey, 1988–1994. Am J Clin Nutr.

[3] Shenkin A (2006). Micronutrients in health and disease. Postgrad Med J.

[4] Toniolo P, Riboli E, Protta F, Charrel M, Cappa AP (1989). Calorie-providing nutrients and risk of breast cancer. J Natl Cancer Inst.

[5] Gandini S, Merzenich H, Robertson C, Boyle P (2000). Meta-analysis of studies on breast cancer risk and diet: the role of fruit and vegetable consumption and the intake of associated micronutrients. Eur J Cancer.

[6] Favero A, Salvini S, Russo A, Parpinel M, Negri E, Decarli A (1997). Sources of macro-and micronutrients in Italian women: results from a food frequency questionnaire for cancer studies. Eur J Cancer Prev.

[7] Wardle J, Parmenter K, Waller J (2000). Nutrition knowledge and food intake. Appetite.

[8] Baranowski T, Cullen KW, Nicklas T, Thompson D, Baranowski J (2003). Are current health behavioral change models helpful in guiding prevention of weight gain efforts?. Obes Res.

[9] Najmabadi S, Nojomi M (2005). Evaluation of micronutrient intakes (vitamins and minerals) in university students. MEDICAL SCIENCES.

[10] James W, Ralph A, Bellizzi M (1997). Nutrition policies in western Europe: national policies in Belgium, the Netherlands, France, Ireland, and the United Kingdom. Nutr Rev.

[11] McGinnis JM, Foege WH (1993). Actual causes of death in the United States. Jama.

[12] Girois SB, Kumanyika SK, Morabia A, Mauger E (2001). A comparison of knowledge and attitudes about diet and health among 35-to 75-year-old adults in the United States and Geneva Switzerland. Am J Public Health..

[13] Azemati B, Heshmat R, Sanaei M, Salehi F, Sadeghi F, Ghaderpanahi M (2013). Nutritional knowledge, attitude and practice of Iranian households and primary health care staff: NUTRIKAP Survey. J Diabetes Metab Disord.

[14] Ahadi Z, Heshmat R, Sanaei M, Shafiee G, Ghaderpanahi M, Homami MR (2014). Knowledge, attitude and practice of urban and rural households towards principles of nutrition in Iran: results of NUTRIKAP survey. J Diabetes Metab Disord.

[15] Barker ME, Thompson KA, McCLEAN SI (1995). Attitudinal dimensions of food choice and nutrient intake. Br J Nutr.

[16] Lennernäs M, Fjellström C, Becker W, Giachetti I, Schmitt A, Remaut dWA (1997). Influences on food choice perceived to be important by nationally-representative samples of adults in the European Union. Eur J Clin Nutr..

[17] Xu L, Ma H, Yang T, Liu T (2004). A cross-sectional study on the changes in dietary behavior stages in resident. Zhonghua Yu Fang Yi Xue Za Zhi.

[18] Ao R, Safarian A, Modaresi J, Poorabdollahi P, Mahdavi R (2010). Effect of nutrition education on nutrition knowledge, attitudes and practices of women in Tabriz University of Medical Sciences. Medical Sciences.

[19] Mowe M, Bosaeus I, Rasmussen HH, Kondrup J, Unosson M, Rothenberg E (2008). Insufficient nutritional knowledge among health care workers?. Clin Nutr.

